# Protective effects of physical activity on mental health outcomes during the COVID-19 pandemic

**DOI:** 10.1371/journal.pone.0279468

**Published:** 2022-12-30

**Authors:** Nikita K. Koziel Ly, Ladan Mohamud, Paul J. Villeneuve, Kimberly Matheson, Hymie Anisman, Melissa J. Chee

**Affiliations:** 1 Department of Neuroscience, Carleton University, Ottawa, Ontario, Canada; 2 Royal Ottawa Institute of Mental Health Research, Ottawa, Ontario, Canada; University of Tsukuba, JAPAN

## Abstract

The COVID-19 pandemic has been linked with increased reports of depression, anxiety, and stress. Stay-at-home directives during the pandemic-imposed lifestyle changes, including eating and sedentary behaviors that can further undermine mental health outcomes. Physical activity is a vital component for metabolic health, as well as for mental health by serving as an active coping strategy to manage stress and promote resilience. Global reports of increased sedentary leisure behaviors have been associated with feelings of depression and anxiety, but it unclear whether the relationship between physical activity and depression or anxiety persists over time. In this longitudinal study, we investigated (i) whether physical activity at the onset of the pandemic was related to feelings of depression or anxiety over time and (ii) whether this relationship was mediated by stress appraisals during the pandemic. We surveyed 319 adults living in Canada or the United States to assess physical activity, stress appraisals, and mental health outcomes at two time points over a 6-month period. We found a reduction in leisure-time physical activity that was linked to subsequent feelings of depression. Furthermore, individuals with lower levels of physical activity were more likely to appraise their COVID-19 situation to be uncontrollable at pandemic onset and as the pandemic continued. Stress appraisals of threat and uncontrollability were also positively related to feelings of depression. Modelling these three factors together showed that appraising a situation as uncontrollable mediated the relationship between initial physical activity and subsequent depressive feelings. Although correlational, these data highlight the protective role of leisure-time physical activity against worsened mental health outcomes during periods of prolonged stress.

## 1 Introduction

The COVID-19 pandemic and the accompanying distress has been reported to foster the development and exacerbation of psychological disturbances, such as anxiety and depression [1, 2]. Several thousand original research articles as well as many meta-analyses and systematic reviews have assessed the multiple factors that moderate the ties between COVID-19 and the occurrence of diverse mental and physical health disturbances. Early in the pandemic, individuals in some segments of society seemed to be particularly distressed (e.g., women caring for young children; individuals who encountered financial loss), and such distress was particularly pronounced among individuals who had been dealing with physical and mental health issues prior to the pandemic [3]. Moreover, enduring cognitive and psychiatric disturbances were also observed among survivors of COVID-19 infection, irrespective of the severity of their illness [4].

As much as the COVID-19 pandemic elicited lasting distress, an extensive analysis revealed that the initial climb in anxiety and depression evident at the start of the pandemic was frequently not sustained, declining to pre-pandemic levels after several months [5]. This may be because individuals may be imbued with features that favor resilience and can adapt in the face of relatively intense stressors [6]. This may entail the way individuals appraise a stressor, which includes the perceived stressfulness of the situation, the threat created, and their ability to cope with the threat [7]. In turn, these appraisals promote the adoption of particular coping methods that can vary with the appraised controllability, predictability, uncertainty, and chronicity of the stressor [8]. In response to certain stressors, the coping strategies endorsed may be ineffective and even counterproductive, for example by the reliance on substance use [9] or eating to cope [10].

One form of coping is the pursuit of physical activity [11]. Ordinarily, physical activity has multiple health benefits, including acting in a prophylactic and therapeutic capacity to diminish mood disorders like depression and anxiety [12], as well as to enhance heart health [13] and act against cancer occurrence [14]. During the pandemic, working from home, job loss, reduced availability of exercise facilities, and reduced social support because of stay-at-home orders were common factors contributing to sedentary behaviors or diminished physical activity [15–17]. Indeed, there was an overall decline in the level of physical activity and concomitant increase of sedentary behaviors [18], which can undermine physical health [19] and mental health [20]. Indeed, during the pandemic period, those who did not exercise tended to report higher feelings of stress, depression, or anxiety [21].

In addition to serving as a coping method, exercise and leisure-time activities may affect appraisal processes (e.g., stressfulness, threat, control by self, and uncontrollability) that could thereby influence mood, as positive stress appraisal style is predictive of resilience [22]. The perception of control can minimize stress responses to a stressful situation [23]. Furthermore, as physical activity is an active coping mechanism [11] and is associated with an internal locus of control where individuals believe they have control over the outcomes of a situation [24], physical activity may be associated with appraisals of controllability. In the present longitudinal investigation, we assessed whether there was a change in physical activity levels; to what extent previous physical activity during leisure time or at the workplace were linked to self-reported depressive or anxiety symptoms over time; and whether these relations were mediated by stress appraisals that individuals made concerning COVID-19. Overall, we found that physical activity during leisure time at pandemic onset was associated with reduced appraisals of uncontrollability regarding continuing COVID-19-related stressors, which predicted lower feelings of depression.

## 2 Materials and methods

### 2.1 Participants and procedure

This study was approved by the Carleton University Research Ethics Board B (Clearance #112909) and written consent was obtained prior to the start of each survey. Participants were recruited between May–June 2020, *via* social media (i.e., Instagram, Twitter, and Facebook), email and snowball sampling, and Amazon Mechanical Turk (MTurk) (Amazon Web Services, Seattle, WA) to assess the effects of a pervasive stressor, like the COVID-19 pandemic, on the relationship between mental and physical health. Eligible participants completed a 30-minute online survey querying their physical activity, mental health, and how they appraise stressful situations. Eligible participants were 18 years of age or older, able to read and understand English, and living in Canada or the United States. Participants recruited *via* MTurk were also required to meet a Human Intelligence Task (HIT) approval rate (i.e., proportion of completed tasks approved by job requesters) equal to or greater than 95%. At the end of the Time 1 (T1) survey, participants were asked if they consented to being contacted in the future to complete the survey a second time. We redistributed the same online survey on physical activity, mental health, and appraisals to consenting participants in Time 2 (T2) between September–October 2020, as it coincided with a rise in active COVID-19 cases in Canada [25] and the United States [26] and delineated a period of prolonged stress.

Datasets of responses collected at each time point were cleaned to exclude participants that selected *No* for response effort (i.e., *I provided honest*, *high-quality answers to the survey questions)*, submitted their responses in 5-minutes or less, did not complete 100% of questions, and/or provided height and weight values that resulted in a Body Mass Index (BMI) less than 15 or greater than 55. The datasets from T1 and T2 were matched so only participants completing both surveys were included, which formed our final sample comprising 319 participants (**Table 1**). Chi-squared (χ^2^) analyses indicated that participants who continued throughout the study and those who dropped out or were excluded after data cleaning did not differ as a function of age, gender, education, income, employment, relationship status, or country of residence. Only participants’ living arrangement was associated with the likelihood of dropping out (χ^2^ = 8.13, p = 0.043). Namely, participants living with others and children (40.7%) or participants living alone with children (75.0%) had higher drop-out rates. Participants in the final sample were predominantly White, female, less than 29 years old (*M* = 37.7 ± 16.2) and had an average BMI of 25.5 ± 5.9 kg/m^2^. Most participants reported a household income over $105,000, had some form of post-secondary education, and were living with others in Canadian urban areas.

**Table 1:** Description of participant characteristics.

Variable	Number of participants (%)		
	Time 1^a^	Time 1^b^	Time 2^b^
**Gender** ^c^			
Female	347 (75.1%)	248 (77.7%)	
Male	104 (22.5%)	66 (20.7%)	
Other (e.g., transgender, non-binary)	11 (2.4%)	5 (1.6%)	
**Cultural affiliation** ^c^			
White and/or Euro-Caucasian	351 (76.0%)	242 (75.9%)	
Black and/or African	23 (5.0%)	14 (4.4%)	
Asian (West, South, East, or Southeast)	68 (14.7%)	65 (20.4%)	
Latin American and Caribbean	24 (5.2%)	12 (3.8%)	
Indigenous	15 (3.2%)	3 (0.9%)	
Other	2 (0.4%)	2 (0.6%)	
**Household income** ^c^			
Under $15,000	28 (6.1%)	12 (3.8%)	
$15,000–$29,999	45 (9.7%)	29 (9.1%)	
$30,000–$44,999	41 (8.9%)	25 (7.8%)	
$45,000–$59,999	53 (11.5%)	34 (10.7%)	
$60,000–$74,999	47 (10.2%)	41 (12.9%)	
$75,000–$89,999	56 (12.1%)	31 (9.7%)	
$90,000–$104,999	47 (10.2%)	32 (10.0%)	
$105,000 or more	145 (31.4%)	112 (35.1%)	
**Education** ^c^			
High school or less	74 (16.0%)	38 (11.9%)	
Post-secondary education	387 (83.8%)	280 (87.8%)	
Other	1 (0.2%)	1 (0.3%)	
**Relationship status**			
Single, not seeing anyone	134 (29.0%)	111 (34.8%)	107 (33.5%)
In a relationship (not married)	111 (24.0%)	58 (18.2%)	57 (17.9%)
Married/Cohabiting	191 (41.3%)	136 (42.6%)	139 (43.9%)
Separated/ Divorced	19 (4.1%)	11 (3.4%)	11 (3.4%)
Widowed	6 (1.3%)	3 (0.9%)	5 (1.6%)
**Employment status**			
Employed Part-Time	90 (19.5%)	59 (18.5%)	69 (21.6%)
Employed Full-Time	205 (44.4%)	116 (36.4%)	116 (36.4%)
Self-employed	29 (6.3%)	27 (8.5%)	26 (8.2%)
Unemployed	95 (20.6%)	75 (23.5%)	67 (21.0%)
Retired	35 (7.6%)	33 (10.3%)	32 (10.0%)
Other	8 (1.7%)	9 (2.8%)	9 (2.8%)
**Location**			
Canada	374 (81.0%)	275 (86.2%)	275 (86.2%)
United States	88 (19.0%)	44 (13.8%)	44 (13.8%)
**Living arrangement**			
Living alone	53 (11.5%)	50 (15.7%)	55 (17.2%)
Living with others but not children	276 (59.7%)	197 (61.8%)	206 (64.6%)
Living with others and children	113 (24.5%)	67 (21.0%)	57 (17.9%)
Living alone and children	20 (4.3%)	5 (1.6%)	1 (0.3%)

^a^ Participants that dropped out after Time 1 or were excluded after data cleaning (*n* = 462).

^b^ Participants in the final sample (*n* = 319).

^c^ Responses queried at Time 1 only.

These surveys were administered using Qualtrics Survey Platform (Provo, UT). Participants accessed the surveys and provided informed consent to voluntarily participate. The surveys included questions to assess demographics, physical activity levels, stress appraisals, and mental health. At the end of each survey, participants received a debriefing form that included contact information and additional resources if they had questions or felt distressed after answering questions related to their mood and ability to cope. Upon each survey completion, participants could choose to receive a $5 CAD electronic gift card to either Amazon.ca, Second Cup, or Starbucks. MTurk participants were compensated ($3 USD) by fund transfer into their MTurk account.

### 2.2 Measures

#### 2.2.1 Demographics

Participants indicated their age, self-reported height and weight, gender, cultural affiliation, relationship status, household income, employment status, national location, living arrangement, and highest level of attained education. We calculated BMI by dividing the weight of the participant in kilograms by the square of their height in meters as a measure of obesity. Participants selecting *Other* as their current employment status included university students and individuals on leave (e.g., maternity, sick, disability). Educational status was determined as the highest level of education completed. Those with high school or less may have no or some high school completed or a high school diploma. Those with post-secondary education may have college/university credit; trade/technical/vocational training; or an Associate, Bachelor’s, Master’s, Professional, or Doctorate degree.

#### 2.2.2 Physical activity

Participants responded to the Stanford Brief Activity Survey (SBAS) [27] to indicate their level of physical activity in the workplace (on-the-job) and during leisure-time. For both job- and leisure-related settings, participants were presented with five statements describing varying intensities of physical activity in increasing order and asked to select the one that was most applicable to them within the past week. Statements for on-the-job physical activity ranged from 1 *(I have no job or regular work)* and 2 *(I spend most of the day sitting or standing)* to 5 *(I spent most of the day doing hard physical labor)*. Statements for leisure-time physical activity ranged from 1 *(Most of my leisure time was spent without very much physical activity)* to 5 *(I engaged in a regular program of physical fitness involving heavy physical activity five to seven times a week)*.

#### 2.2.3 Appraisals

To assess participants’ thoughts on various aspects of their situation during the COVID-19 pandemic at each time point, the 28-question Stress Appraisal Measure (SAM) [28] was used. Participants rated on a five-point scale ranging from 1 (not at all) to 5 (extremely) how they viewed their situation at the time of survey completion (e.g., *is this a totally hopeless situation*?). These scores were averaged to obtain seven dimensions of stress appraisals: threat, challenge, centrality, controllable by self, controllable by others, uncontrollable, and stressfulness (Cronbach’s *α* for threat = 0.81; challenge = 0.70; centrality = 0.89; control by self = 0.89; control by others = 0.92; uncontrollable = 0.79; and stressfulness = 0.85).

#### 2.2.4 Mental health

The 21-item Depression, Anxiety, and Stress Scale (DASS21) [29, 30] assessed mental health at each time point. Participants were presented with 21 statements describing various emotional states (i.e., *I found it hard to wind down*, *I was aware of dryness of my mouth*, etc.) and indicated on a four-point scale how much each statement applied to them in the past week with 1 being *did not apply to me* and 4 being *applied to me very much*. The scale was divided into three subscales, depression, anxiety, and stress, and the score for each subscale was calculated as the sum of scores from seven statements per subscale multiplied by 2 [29] (Cronbach’s *α* for depression = 0.92; anxiety = 0.84; stress = 0.89).

### 2.3 Statistics

All statistical analyses were completed using IBM SPSS v27.0 (IBM, Armonk, NY).

Chi-squared (χ^2^) analyses were used to identify possible differences in T1 demographic characteristics between participants in the final dataset and those who dropped out or excluded after data cleaning.

A repeated-measures two-way analysis of variance (ANOVA) was performed to assess the effects of time and activity (i.e., on-the-job or during leisure time) on physical activity scores. Significant main effects were followed up with simple effect analyses using a repeated-measures one-way ANOVA.

To determine whether physical activity at T1 was associated with appraisals (at T1 or T2) and mental health outcomes (at T2), we performed bivariate Pearson correlations. Only variables related to physical activity, specifically leisure-time physical activity, were pursued further.

To determine the relationship between selected appraisals and depression, we performed bivariate Pearson correlations. As all selected appraisals at both T1 and T2 were significantly correlated with depression in T2 (*p* < 0.05), hierarchical regression analyses were used to separately assess the impact of each T1 appraisal on T2 depression (first step) and of T1 and T2 appraisals on T2 depression (final step).

After examining correlations to determine whether physical activity and appraisals were related to mental health outcomes, and whether physical activity and appraisals were related to each other, mediation analyses were conducted to model the cumulative relationship among all three variables. PROCESS macro v4.0 Model 4 [31] was applied wherein leisure-time physical activity at T1 was the predictor; appraisals (threatening, controllable by self, uncontrollable) at T2 were the mediators; T1 appraisals and T1 depression were the covariates; and depression at T2 was the outcome variable. The moderating effect of demographic variables were then assessed using PROCESS macro Model 8 (S1 Fig) [31].

Comparisons resulting in a two-sided *p* < 0.05 were considered to be statistically significant. Significance thresholds are indicated in figure legends.

## 3 Results

### 3.1 Shift in intensity of leisure-time physical activity

**Fig 1:**
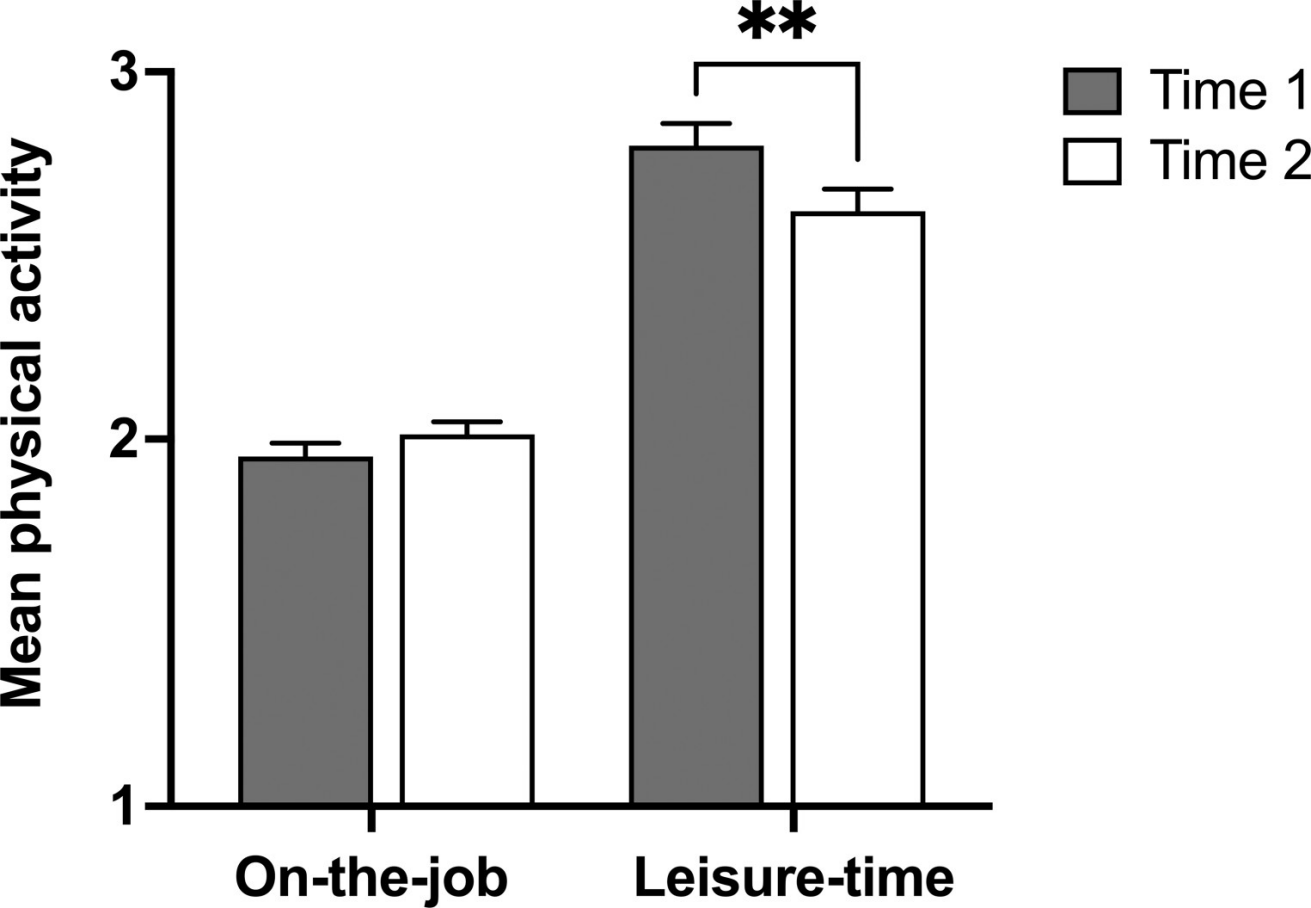
Leisure-time physical activity decreased as the pandemic continued. Differences in mean on-the-job *versus* leisure-time physical activity scores between Time 1 (May–June 2020) and Time 2 (September–October 2020). Data shown as mean ± SEM; n = 319. One-way repeated measures ANOVA: **, *p* < 0.01.

To determine if there was a shift in physical activity levels between the early and later stage of the COVID-19 pandemic, we compared activity levels reported in the workplace and during times of leisure at T1 and T2. There was an overall decrease in physical activity from T1 (2.93 ± 0.05, n = 319) to T2 (2.75 ± 0.05, n = 319; df = 31, *p* = 0.001) (S1 Table). There was a main effect of activity (*η*^2^ = 0.340, *F*(1,318) = 163.90, *p* < 0.001), as leisure activity scores were higher than work-related activity scores at both time points. There was no main effect of time (*η*^2^ = 0.009, *F*(1,318) = 3.03, *p* = 0.083), but there was a significant Time × Activity interaction (*η*^2^ = 0.025, *F*(1,318) = 8.16, *p* = 0.005). Simple effects analyses indicated a decrease in leisure-time physical activity over time (T1: 2.80 ± 0.06; T2: 2.62 ± 0.06; *η*^2^ = 0.024, *F*(1,318) = 7.81, *p* = 0.006), but not on-the-job physical activity (T1: 1.95 ± 0.04; T2: 2.01 ± 0.04; *η*^2^ = 0.006, *F*(1,318) = 2.04, *p* = 0.154) (Fig 1).

### 3.2 Physical activity associated with appraisals and mental health outcomes

Physical activity can serve as a coping strategy [11], thus we determined whether leisure-time physical activity was associated with how participants appraised stressful situations (S2 Table). As shown in Table 2, work-related physical activity at T1 was not related to T1 stress appraisals. However, participants engaging in leisure-time physical activity at T1 were more likely to appraise a situation to be controllable by oneself and less likely to appraise a situation as uncontrollable. Interestingly, T1 leisure-time physical activity predicted future appraisals of stressful situations. In addition to higher appraisal of control by oneself and lower appraisal of uncontrollability at T2, initial leisure-time physical activity at T1 decreased the likelihood of appraising a situation as threatening later at T2. Leisure-time physical activity was also predictive of mental health outcomes (Table 3). Namely, higher T1 leisure-time physical activity was related to lower T1 feelings of depression and anxiety, but not to feelings of stress. As the link between leisure-time physical activity and concurrent mental health outcomes is well-established [20], we assessed whether the benefits of leisure activity can be extended to predicting mental health outcomes over time. The relationship between T1 leisure-time physical activity and mental health was also observed for T2 feelings of depression, but not anxiety or stress. Taken together, these relationships suggested that actively choosing to engage in physical activity in one’s leisure time was positively associated with stress appraisals and lower depressive feelings.

**Table 2:** Pearson correlations between physical activity at Time 1 and appraisals at Time 1 and Time 2 (*n* = 319).

	Appraisal	Physical activity at Time 1	
		On-the-job	Leisure-time
Time 1	Threat	–0.03	–0.05
	Challenge	–0.004	0.09
	Centrality	–0.01	–0.01
	Control-by-self	0.03	0.16 **
	Control-by-others	0.06	0.11
	Uncontrollable	0.02	–0.14 **
	Stressful	0.003	–0.07
Time 2	Threat	0.000	–0.11 *
	Challenge	–0.09	0.11
	Centrality	–0.05	–0.05
	Control-by-self	0.03	0.16 **
	Control-by-others	0.15 **	0.08
	Uncontrollable	–0.02	–0.19 **
	Stressful	0.01	–0.07

* *p* < 0.05

** *p* < 0.01.

**Table 3:** Pearson correlations between physical activity at Time 1 and mental health at Time 1 and Time 2 (*n* = 319).

	Mental health	Physical activity at Time 1	
		On-the-job	Leisure-time
Time 1	Depression	–0.06	–0.18 **
	Anxiety	0.03	–0.11 *
	Stress	0.003	–0.11
Time 2	Depression	–0.09	–0.12 *
	Anxiety	0.02	–0.06
	Stress	0.004	–0.06

* *p* < 0.05.

As the appraisal and mental health correlations occurred more frequently or exclusively with leisure-time physical activity, and as ANOVA showed leisure-time physical activity but not on-the-job physical activity significantly differing from T1 to T2, only leisure-time physical activity in T1 was used going forward. Furthermore, since leisure-time physical activity was associated only with feelings of depression at both times points, only this relationship was selected for subsequent analyses. Similarly, only appraisals (i.e., threat, control-by-self, and uncontrollable) that were related to leisure-time physical activity were pursued further.

### 3.3 Appraisals associated with mental health outcomes

**Fig 2:**
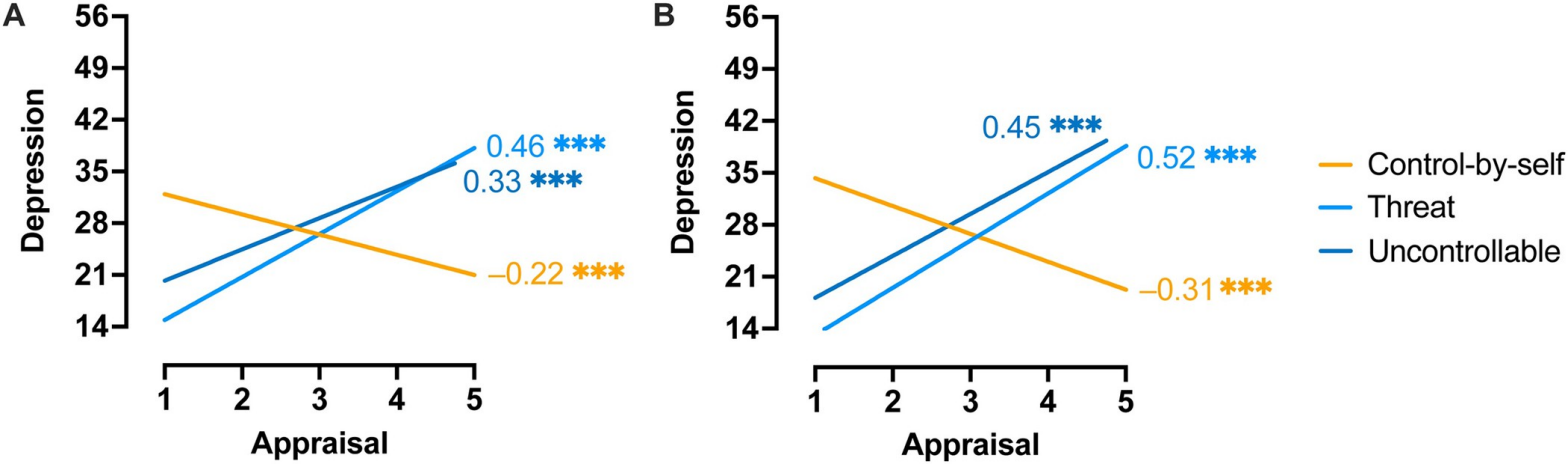
Appraisals of stressful situations predicted feelings of depression. Relationship between threat (light blue), uncontrollable (dark blue), and controllable-by-oneself (orange) appraisals at Time 1 (**A**) and Time 2 (**B**) with depression at Time 2. Lines on the plot represent simple linear regressions, and numerals represent bivariate Pearson correlations, r: ***, *p* < 0.001.

As leisure-time physical activity was related to stress appraisals and feelings of depression, we assessed whether appraisals and feelings of depression were related to each other [32, 33]. Appraisals of threat and uncontrollability in both T1 (Fig 2A) and T2 (Fig 2B) were associated with greater feelings of depression at T2, while appraising a situation to be controllable was associated with lower feelings of depression (Fig 2).

Since appraisals at both time points were related to feelings of depression at T2, we performed hierarchical regression analyses to determine whether appraisals at one time point (T1 or T2) contributed more to these feelings of depression than the other. Though T1 appraisals were a significant predictor of depression (*R*^*2*^ = 0.234, *F*(3, 315) = 32.03, *p* < 0.001), T2 appraisals contributed to T2 depression over-and-above appraisals made earlier in the pandemic (*R*^*2*^_*change*_ = 0.128, *F*_*change*_(3, 312) = 20.85, *p* < 0.001). Appraisals of threat, control-by-self, and uncontrollability made in later stages of the pandemic each accounted for unique variance in depression, as seen in Table 4. The strongest predictor of depression at both T1 and T2 was appraisals of the situation as threatening.

**Table 4:** Regression coefficients for appraisals at Times 1 and 2 predicting depression at Time 2 based on final step statistics.

Model		Depression at Time 2			
		*b*	*se*	*Beta*	*r*
Time 1	Control-by-self	0.47	0.74	0.04	–0.22 **
	Threat	2.15	0.87	0.17 *	0.46 **
	Uncontrollable	0.02	0.74	0.002	0.33 **
Time 2	Control-by-self	–2.47	0.71	–0.21 **	–0.31 **
	Threat	3.01	0.84	0.25 ***	0.52 **
	Uncontrollable	2.70	0.72	0.21 ***	0.45 **

*b*, unstandardized regression coefficient; *se*, standard error; *Beta*, standardized coefficient; *r*, zero-order correlation.

* *p* < 0.05

** *p* < 0.01

*** *p* < 0.001.

As T2 appraisals had a greater contribution to the model, they were input in subsequent mediation models as mediators and T1 appraisals were input as covariates.

### 3.4 Lower leisure activity predict later feelings of depression when encountering situations appraised as uncontrollable

**Fig 3:**
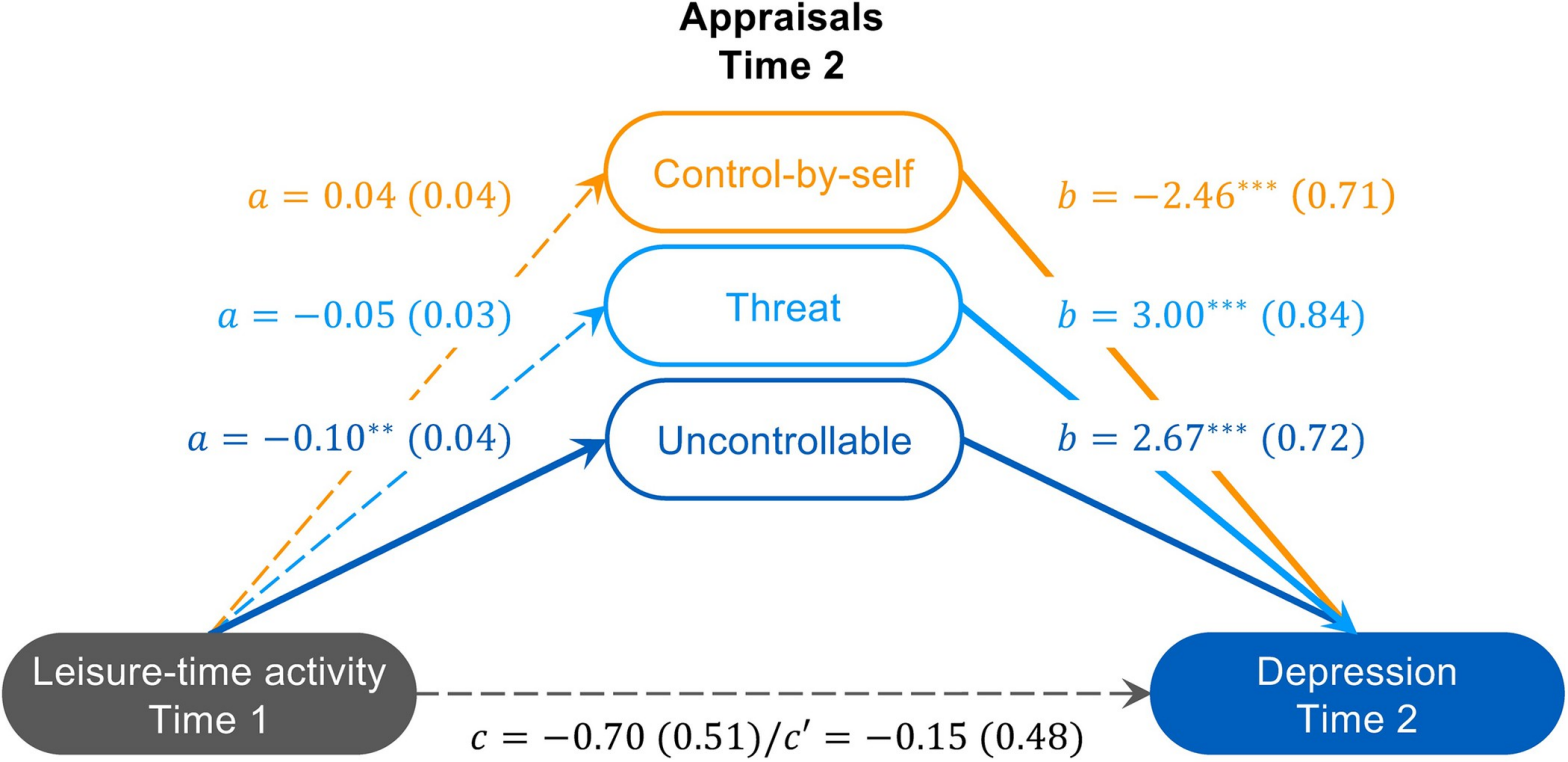
Uncontrollability linked leisure-time physical activity with subsequent feelings of depression. Model (unstandardized coefficients (*se*)) of the relationship between leisure-time physical activity at Time 1 and feelings of depression at Time 2 as mediated through appraisals of threat, control-by-self, and uncontrollable at Time 2. Covariates were appraisals of threat, control by self, and uncontrollable at Time 1. Total effect pathway (*c*), direct effect pathway (*c*’), indirect effect pathway (*a*, *b*). ** *p* < 0.01; *** *p* < 0.001.

Having observed that initial leisure-time physical activity was related to feelings of depression (Table 3), which was associated with how a participant appraised their present situation (Table 4), we performed an analysis to determine whether the relationship between physical activity and later depressive symptoms was mediated by later appraisals. As shown in Fig 3, T1 leisure-time activity was a significant predictor of uncontrollability appraisals at T2, but it was not a significant predictor of threat or control-by-self appraisals at T2. By contrast, T2 appraisal of threat, control-by-self, and uncontrollable predicted T2 feelings of depression.

T1 leisure-time physical activity was not a direct predictor of T2 depressive feelings, but mediated models were significant when appraisals, in particular appraisals of uncontrollability were considered (*Effect* = –0.28 (*se* = 0.13), CI_.95_ [–0.56, –0.06]). Mediation through appraisals of threat (*Effect* = –0.16 (*se* = 0.11), CI_.95_ [–0.39, 0.04]) or control-by-self were not significant (*Effect* = –0.11 (*se* = 0.10), CI_.95_ [–0.32, 0.07]). The demographic variables of gender (S3 Table), age (S4 Table), BMI (S5 Table), and employment status (S6 Table) were assessed as moderators in our moderated mediation analyses (see S1 Fig) and found to be not significant.

Importantly, the same relationships were apparent when controlling for T1 depression (*c* = 0.02 (*se* = 0.44), *p* = 0.964; *c’* = 0.40 (*se* = 0.42), *p* = 0.342; *Control-by-self*: *a* = 0.03 (*se* = 0.04), *p* = 0.399; *b* = –1.95 (*se* = 0.61), *p* = 0.002; *effect* = –0.06 (*se* = 0.08), CI_.95_ [–0.23, 0.09]; *Threat*: *a* = –0.04 (*se* = 0.03), *b* = 2.21 (*se* = 0.73), *p* = 0.003; *p* = 0.239; *effect* = –0.10 (*se* = 0.08), CI_.95_ [–0.26, 0.06]; *Uncontrollable*: *a* = –0.09 (*se* = 0.04), *p* = 0.018; *b* = 2.37 (*se* = 0.63), *p* < 0.001; *effect* = –0.22 (*se* = 0.11), CI_.95_ [–0.47, –0.04]).

## 4 Discussion

Physical activity can promote stress resilience [34] and serve as an effective coping strategy [11], including during the pandemic [35–38]. It is also effective at diminishing depressive symptoms as well as symptoms of other mental illnesses [39]. In our longitudinal study, we detected a decrease in leisure-time physical activity over a 6-month period from the beginning of the pandemic. Importantly, engaging in lower amounts of leisure-time physical activity initially was inversely related to feelings of depression over time. Individuals who engaged in more physical activity in their leisure time were more likely to appraise their COVID-19 situation to be within their control. Interestingly, leisure-time physical activity at the start of the pandemic was also accompanied by participants appraising that their situation was controllable and less threatening as the pandemic progressed; such appraisals in turn predicted lower depressive symptoms later in the pandemic.

The vast majority of studies comparing physical activity before and during the pandemic reported reduced physical activity levels [40], and this was also reflected by worldwide reports of reduced spontaneous activity during the pandemic [41, 42]. We extended this observation by comparing physical activity levels over the course of the pandemic between May and October 2020. There was an overall 7% decrease in physical activity that was ascribed to reduced leisure- but not job-related physical activity. More participants reported sedentary behavior or little to no engagement in physical activity for leisure as the pandemic progressed. Similarly, fewer participants engaged in a regular high intensity fitness program. This was consistent with studies comparing activity levels before and at the start of the pandemic. For example, individuals spent more time engaging in light physical activity like walking but less time engaged in vigorous physical activity [18].

Reduced physical activity during the pandemic can negatively impact concurrent mood and feelings of depression or anxiety [43]. We found that low leisure-time physical activity had a lasting relationship to mental health outcomes even with prolonged exposure to a stressor. Our findings are consistent with the view that physical activity may alleviate depressive symptoms [44], but the inclusion of physical activity need not be of high intensity nor for a prolonged duration, as frequent low-intensity activity was accompanied by the most sustained benefits [45, 46]. It is particularly notable that exercise measured at Time 1 predicted diminished symptoms of depression assessed at Time 2. As such, it might be assumed that a causal connection exists between the two. However, individuals who regularly engage in exercise might also adopt other positive health behaviors (e.g., low inflammatory diet), which may contribute to other long-term benefits related to depression.

The mechanisms underlying the actions of physical activity are uncertain given the wide array of biological change introduced by exercise. Physical activity promotes the release of β-endorphins that bind to brain opiate receptors, but the effect of β-endorphins may result in positive or negative experiences because of moderate or high exercise intensity, respectively [47]. Exercise can also release other chemical messengers like noradrenalin, dopamine, and serotonin that play an important part in regulating mood [48]. Indeed, these monoaminergic systems have been targeted in anti-depressant medications, though non-monoamine approaches are now emerging [49]. As well, moderate exercise may diminish circulating inflammatory factors [50], whereas intense exercise was accompanied by elevated presence of inflammatory factors that have been linked to depressive symptoms [51]. As systemic inflammation has been associated with specific symptoms of depression [52], it is reasonable to hypothesize that these processes may account for the benefits derived from exercise. When exercise was accompanied by a diet that was low in calories, inflammation was reduced, and this was accompanied by improved signs associated with illnesses, such as type 2 diabetes and coronary artery disease [53]. A combination of diet and exercise likewise had superior effects in diminishing depressive symptoms relative to either treatment alone [54].

The relationship between leisure-time physical activity and diminished feelings of depression may reflect increased resilience emanating from indirect effects accrued with exercise. Resilience involves a network of relationships between the stressor and their characteristics, appraisals of the stressor, coping strategy, and reassurances of safety [33]. As in the case of other chronic stressors, resilience factors may help adapt to the pressures and distress experienced during the COVID-19 pandemic [6]. During the pandemic, resilience was linked to lifestyles that may be family-centered and that includes outdoor activities or exercise [34]. Furthermore, physical activity promotes resilience against psychosocial stressors [55, 56], and was ascribed to broad and diverse psychosocial features, including optimism [57, 58], perceived social supports [22], and positive stress appraisal styles [22]. We found that leisure-time physical activity favored appraisals of the COVID-19 situation as less threatening, less uncontrollable, and more controllable by oneself. This is consistent with the perceived control imparted by engaging in physical activity [24], which is used to cope with the pandemic situation [11].

Interestingly, appraising the situation to be uncontrollable, but not control-by-self or threatening, linked initial leisure-time physical activity with feelings of depression six months later. There may have been broadly held feelings of uncontrollability and uncertainty regarding most aspects of life during the early phases of the pandemic (e.g., will vaccines be available, how long will a lockdown last, etc.). The unpredictability of the pandemic also presented ambiguity, which is more likely to link control appraisals to locus of control [28]. The mediating role of uncontrollability may be related to a shift towards external locus of control [59], which corresponds to perceptions that the outcome of a situation is due to fate rather than their action or decision [60]. This external locus of control may render participants to be more susceptible to the impact of the pandemic [61]. Indeed, external locus of control is positively correlated with feelings of depression [62], and interestingly, adherence to exercise has also been shown to be linked to external locus of control [63]. Overall, the shift away from internal control and deficient coping resources like the pursuit of leisure-time physical activity may also erode resilience to prolonged stressors presented by the pandemic, as resilience has also been shown to mediate the relationship between physical activity and mental health [64].

### 4.1 Limitations

It may not be entirely surprising that variables measured at the same time point (i.e., stress appraisals and depression at Time 2) may be bidirectionally related to each other. However, these findings are still valuable, particularly as appraisal of uncontrollability selectively mediated the relationship between leisure-time activity and depression. Moreover, this was apparent even when controlling for Time 1 appraisals of threat, control-by-self, and uncontrollability. Furthermore, these relations were not moderated by gender, age, or employment status. A more elegant analysis to model the predictive power of leisure-time activity on depression over time could potentially have been provided by analyses, such as that adopted by Little et al. [65] or Ohtani et al. [66]; however, these methods have limited applicability given the absence of a third timepoint to include in the analysis.

We preferred established questionnaires that were brief to minimize the overall length of our survey and improve participant enrollment and completion. For this reason, we chose SBAS to capture overall physical activity, but a more comprehensive physical activity assessment [67] may provide more nuanced insights into the type, frequency, and intensity of physical activity pursued. Indeed, a review of the pertinent literature indicated that greater time engaged in moderate to physical activity was accompanied by reduced anxiety and depression during the COVID-19 pandemic [68].

Several aspects of the investigation also limited the conclusions that could be drawn. Participants were self-selected and hence might not have formed a representative sample, and their responses comprised of retrospective self-reports that may be biased by any number of earlier experiences. Furthermore, the data were correlational thus precluding causal conclusions about the ties between exercise and depression. For instance, while exercise may act against depression, it is equally likely that individuals with depression were less likely to engage in recreational exercise.

The number of participants at the start of the study was moderate, and a substantial portion of participants dropped out between the test sessions. However, with the exception of living arrangement (e.g., living with others and children, living alone with children), demographic variables were not linked to attrition. It is uncertain why attrition was higher in participants living with children, though it is possible that these participants were busier, more stressed or anxious, and hence more likely to drop out.

## 5 Conclusions

Owing to the diverse health benefits that accrue, ‘exercise as medicine’ has become a frequent refrain. The present study underscored the important role of leisure-time physical activity to support mental health during the chronic strain produced by the COVID-19 pandemic. Specifically, our study showed that engaging in activities at higher frequency and intensity was more protective of mental health. However, it would be important to note that positive effects of physical activity have also been reported with frequent but short duration low-intensity exercise like a 30-min walk 3–5 times a week [45]. Our findings indicated that engaging in leisure-time physical was accompanied by diminished depressive symptoms over time, which were mediated by appraisals of the stressor situation.

## Supporting information

S1 Fig Schematic of moderated mediation to assess the moderating role of demographic variables. Model of the relationship between leisure-time physical activity at Time 1 and feelings of depression at Time 2 as mediated through appraisals of threat, control-by-self, and uncontrollable at Time 2. Demographic variables gender, age, BMI, and employment status were assessed as moderators individually. Covariates were appraisals of threat, control by self, and uncontrollable at Time 1.
(TIF)

S1 Table Descriptive statistics of physical activity, stress appraisals, and mental health outcomes at Time 1 and Time 2. (DOCX)

S2 Table Correlation matrix for physical activity, stress appraisals, and mental health at Time 1 and Time 2. (DOCX)

S3 Table Moderating effect of gender on mediation analyses. (DOCX)

S4 Table Moderating effect of age on mediation analyses. (DOCX)

S5 Table Moderating effect of body mass index (BMI) on mediation analyses. (DOCX)

S6 Table Moderating effect of employment status on mediation analyses. (DOCX)
