# Supplementary figures and images for: Protective effects of physical activity on mental health outcomes during the COVID-19 pandemic
Source: PLoS One. 2022 Dec 30;17(12):e0279468. doi: 10.1371/journal.pone.0279468 (PMC9803281; doi:10.1371/journal.pone.0279468)

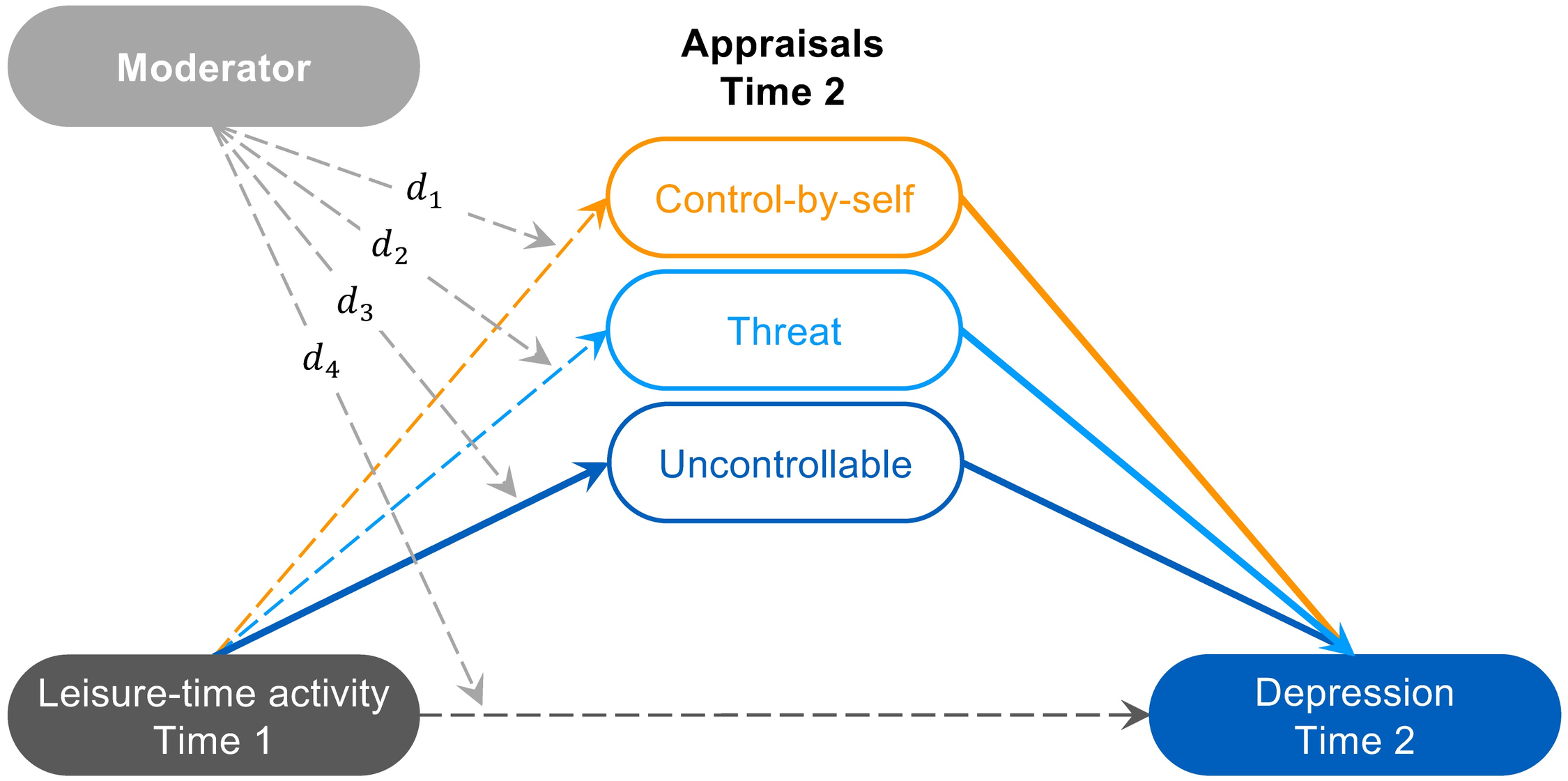

Supplement: S1 Fig — Model of the relationship between leisure-time physical activity at Time 1 and feelings of depression at Time 2 as mediated through appraisals of threat, control-by-self, and uncontrollable at Time 2. Demographic variables gender, age, BMI, and employment status were assessed as moderators individually. Covariates were appraisals of threat, control by self, and uncontrollable at Time 1. (TIF) [file pone.0279468.s001.tif]
