# Supplementary material for: Protective effects of physical activity on mental health outcomes during the COVID-19 pandemic
Source: PLoS One. 2022 Dec 30;17(12):e0279468. doi: 10.1371/journal.pone.0279468 (PMC9803281; doi:10.1371/journal.pone.0279468)
Supplement: S1 Table — (DOCX) [file pone.0279468.s002.docx]

| **S1 Table. Descriptive statistics of physical activity, stress appraisals, and mental health outcomes at Time 1 and Time 2.** | | | | |
| --- | --- | --- | --- | --- |
| **Variable** | **Time 1** | | **Time 2** | |
|  | Mean | *se* | Mean | *se* |
| **Physical activity** |  |  |  |  |
| On-the-job | 1.95 | 0.04 | 2.01 | 0.04 |
| Leisure-time | 2.80 | 0.06 | 2.62 | 0.06 |
| **Appraisal** |  |  |  |  |
| Threat | 2.77 | 0.05 | 2.91 | 0.05 |
| Challenge | 2.61 | 0.05 | 2.55 | 0.05 |
| Centrality | 3.21 | 0.05 | 3.31 | 0.05 |
| Control-by-self | 3.46 | 0.05 | 3.39 | 0.05 |
| Control-by-others | 3.27 | 0.05 | 3.21 | 0.06 |
| Uncontrollable | 2.20 | 0.05 | 2.26 | 0.05 |
| Stressful | 2.84 | 0.05 | 3.00 | 0.05 |
| **Mental health** |  |  |  |  |
| Depression | 24.97 | 0.57 | 25.29 | 0.61 |
| Anxiety | 20.49 | 0.42 | 21.02 | 0.44 |
| Stress | 27.39 | 0.58 | 27.86 | 0.58 |
| *se*, standard error  *n* = 319 in Time 1 and Time 2 | | | | |
