# Supplementary material for: Protective effects of physical activity on mental health outcomes during the COVID-19 pandemic
Source: PLoS One. 2022 Dec 30;17(12):e0279468. doi: 10.1371/journal.pone.0279468 (PMC9803281; doi:10.1371/journal.pone.0279468)
Supplement: S2 Table — (DOCX) [file pone.0279468.s003.docx]

| **Table S2. Correlation matrix for physical activity, stress appraisals, and mental health at Time 1 and Time 2.** | | | | | | | | | | | | | | |
| --- | --- | --- | --- | --- | --- | --- | --- | --- | --- | --- | --- | --- | --- | --- |
|  |  |  | **Time 1** | | | | | | | | | | | |
|  |  |  | **Physical activity** | | **Appraisals** | | | | | | | **Mental health** | | |
|  |  |  | On-the-job | Leisure-time | Threat | Challenge | Centrality | Control-by-self | Control-by-others | Uncontrollable | Stressful | Depression | Anxiety | Stress |
| **Time 2** | **Physical activity** | On-the-job | 0.32 ** | 0.13 * | –0.06 | 0.09 | 0.03 | 0.09 | 0.01 | –0.09 | –0.02 | –0.11 * | –0.05 | –0.04 |
|  |  | Leisure-time | 0.09 | 0.43 ** | –0.21 ** | 0.08 | –0.13 * | 0.11 | –0.05 | –0.20 ** | –0.19 ** | –0.22 ** | –0.16 ** | –0.19 ** |
|  | **Appraisal** | Threat | 0.00 | –0.11 * | 0.70 ** | –0.08 | 0.53 ** | –0.23 ** | –0.12 * | 0.42 ** | 0.57 ** | 0.48 ** | 0.49 ** | 0.52 ** |
|  |  | Challenge | –0.09 | 0.11 | –0.06 | 0.58 ** | 0.10 | 0.37 ** | 0.21 ** | –0.09 | 0.02 | –0.14 * | –0.05 | 0.003 |
|  |  | Centrality | –0.05 | –0.05 | 0.57 ** | 0.10 | 0.65 ** | –0.08 | –0.04 | 0.33 ** | 0.58 ** | 0.42 ** | 0.39 ** | 0.50 ** |
|  |  | Control-by-self | 0.03 | 0.16 ** | –0.19 ** | 0.44 ** | –0.01 | 0.63 ** | 0.39 ** | –0.16 ** | –0.10 | –0.28 ** | –0.20 ** | –0.16 ** |
|  |  | Control-by-others | 0.15 ** | 0.08 | –0.08 | 0.32 ** | –0.01 | 0.41 ** | 0.54 ** | –0.08 | –0.01 | –0.15 ** | –0.04 | –0.07 |
|  |  | Uncontrollable | –0.02 | –0.19 ** | 0.42 ** | –0.03 | 0.34 ** | –0.12 * | –0.06 | 0.48 ** | 0.33 ** | 0.33** | 0.32 ** | 0.38 ** |
|  |  | Stressful | 0.01 | –0.07 | 0.63 ** | 0.02 | 0.57 ** | –0.17 ** | –0.07 | 0.36 ** | 0.66 ** | 0.51 ** | 0.50 ** | 0.60 ** |
|  | **Mental health** | Depression | –0.09 | –0.12 * | 0.46 ** | –0.10 | 0.34 ** | –0.22 ** | –0.12 * | 0.33 ** | 0.37 ** | 0.65 ** | 0.52 ** | 0.55 ** |
|  |  | Anxiety | 0.02 | –0.06 | 0.46 ** | –0.09 | 0.35 ** | –0.16 ** | –0.11 | 0.21 ** | 0.42 ** | 0.56 ** | 0.70 ** | 0.61 ** |
|  |  | Stress | 0.004 | –0.06 | 0.49 ** | –0.04 | 0.41 ** | –0.16 ** | –0.09 | 0.30 ** | 0.48 ** | 0.48 ** | 0.54 ** | 0.71 ** |
| * *p* < 0.05; ** *p* < 0.01 | | | | | | | | | | | | | | |
