# Supplementary material for: Protective effects of physical activity on mental health outcomes during the COVID-19 pandemic
Source: PLoS One. 2022 Dec 30;17(12):e0279468. doi: 10.1371/journal.pone.0279468 (PMC9803281; doi:10.1371/journal.pone.0279468)
Supplement: S3 Table — (DOCX) [file pone.0279468.s004.docx]

| **Table S3. Moderating effect of gender on mediation analyses.** | | | | | | |
| --- | --- | --- | --- | --- | --- | --- |
|  | **Highest order unconditional interaction** | | | **Index of moderated mediation** | | |
|  | *R^2^_change_* | *F_change_* | *p* | *Index* | *se* | CI_.95_ |
| $d_{1}$ | 0.005 | 2.77 | 0.10 | 0.358 | 0.25 | –0.05, 0.91 |
| $d_{2}$ | 0.002 | 0.93 | 0.34 | –0.231 | 0.28 | –0.87, 0.29 |
| $d_{3}$ | 0.000 | 0.01 | 0.92 | –0.026 | 0.26 | –0.55, 0.53 |
| $d_{4}$ | 0.000 | 0.15 | 0.70 |  |  |  |
| *se,* bootstrapped standard error; CI_.95_, bootstrapped 95% confidence interval. | | | | | | |
