# Supplementary material for: Protective effects of physical activity on mental health outcomes during the COVID-19 pandemic
Source: PLoS One. 2022 Dec 30;17(12):e0279468. doi: 10.1371/journal.pone.0279468 (PMC9803281; doi:10.1371/journal.pone.0279468)
Supplement: S4 Table — (DOCX) [file pone.0279468.s005.docx]

| **Table S4. Moderating effect of age on mediation analyses.** | | | | | | |
| --- | --- | --- | --- | --- | --- | --- |
|  | **Highest order unconditional interaction** | | | **Index of moderated mediation** | | |
|  | *R^2^_change_* | *F_change_* | *p* | *Index* | *se* | CI_.95_ |
| $d_{1}$ | 0.000 | 0.12 | 0.73 | 0.002 | 0.01 | –0.01, 0.02 |
| $d_{2}$ | 0.002 | 0.99 | 0.32 | –0.006 | 0.01 | –0.02, 0.01 |
| $d_{3}$ | 0.001 | 0.23 | 0.63 | –0.003 | 0.01 | –0.02, 0.01 |
| $d_{4}$ | 0.002 | 1.21 | 0.27 |  |  |  |
| *se,* bootstrapped standard error; CI_.95_, bootstrapped 95% confidence interval. | | | | | | |
