# Supplementary material for: Protective effects of physical activity on mental health outcomes during the COVID-19 pandemic
Source: PLoS One. 2022 Dec 30;17(12):e0279468. doi: 10.1371/journal.pone.0279468 (PMC9803281; doi:10.1371/journal.pone.0279468)
Supplement: S5 Table — (DOCX) [file pone.0279468.s006.docx]

| **Table S5. Moderating effect of body mass index (BMI) on mediation analyses.** | | | | | | |
| --- | --- | --- | --- | --- | --- | --- |
|  | **Highest order unconditional interaction** | | | **Index of moderated mediation** | | |
|  | *R^2^_change_* | *F_change_* | *p* | *Index* | *se* | CI_.95_ |
| $d_{1}$ | 0.004 | 2.14 | 0.14 | –0.022 | 0.02 | –0.06, 0.01 |
| $d_{2}$ | 0.001 | 0.33 | 0.57 | –0.010 | 0.02 | –0.05, 0.02 |
| $d_{3}$ | 0.002 | 0.93 | 0.33 | –0.017 | 0.02 | –0.05, 0.02 |
| $d_{4}$ | 0.001 | 0.50 | 0.48 |  |  |  |
| *se,* bootstrapped standard error; CI_.95_, bootstrapped 95% confidence interval. | | | | | | |
