# Supplementary material for: Protective effects of physical activity on mental health outcomes during the COVID-19 pandemic
Source: PLoS One. 2022 Dec 30;17(12):e0279468. doi: 10.1371/journal.pone.0279468 (PMC9803281; doi:10.1371/journal.pone.0279468)
Supplement: S6 Table — (DOCX) [file pone.0279468.s007.docx]

| **Table S6. Moderating effect of employment status on mediation analyses.** | | | | | | |
| --- | --- | --- | --- | --- | --- | --- |
|  | **Highest order unconditional interaction** | | | **Index of moderated mediation** | | |
|  | *R^2^_change_* | *F_change_* | *p* | *Index* | *se* | CI_.95_ |
| $d_{1}$ | 0.004 | 2.24 | 0.14 | –0.094 | 0.07 | –0.24, 0.01 |
| $d_{2}$ | 0.003 | 2.03 | 0.16 | –0.100 | 0.08 | –0.27, 0.04 |
| $d_{3}$ | 0.001 | 0.55 | 0.46 | 0.054 | 0.08 | –0.11, 0.21 |
| $d_{4}$ | 0.001 | 0.42 | 0.52 |  |  |  |
| *se,* bootstrapped standard error; CI_.95_, bootstrapped 95% confidence interval. | | | | | | |
